# 2-[(Adamantan-1-yl)amino­meth­yl]-4-chloro­phenol hemihydrate

**DOI:** 10.1107/S1600536812045175

**Published:** 2012-11-07

**Authors:** Xu-Dong Jin, Xue-Yue Yin, Lu-Sha Xu, Chun-Hua Ge, Xiao-Hong Chang

**Affiliations:** a College of Chemistry, Liaoning University, Shenyang 110036, People’s Republic of China

## Abstract

In the title compound, C_17_H_22_ClNO·0.5H_2_O, the water mol­ecule O atom resides on a twofold rotation axis. In the organic mol­ecule, the phenol group forms an intra­molecular O—H⋯N hydrogen bond. In the crystal, pairs of organic mol­ecules are hydrogen bonded through bridging solvent water mol­ecules, forming chains along the *b*-axis direction.

## Related literature

For the synthesis and crystal structure of 2-[(adamantan-1-yl­amino)­meth­yl]phenol, see: Wang & Tao (2012 ▶). For the synthesis and applications of amantadine derivatives, see: Camps *et al.* (2008 ▶).
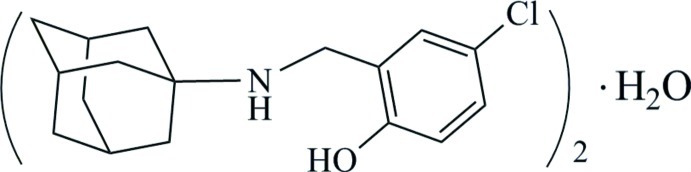


## Experimental

### 

#### Crystal data

C_17_H_22_ClNO·0.5H_2_O
*M* *_r_* = 300.82
Monoclinic, 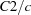

*a* = 25.469 (16) Å
*b* = 6.365 (4) Å
*c* = 18.306 (11) Å
β = 91.815 (12)°
*V* = 2966 (3) Å^3^
*Z* = 8
Mo *K*α radiation
μ = 0.26 mm^−1^
*T* = 296 K
0.35 × 0.30 × 0.16 mm

#### Data collection

Bruker APEXII CCD area-detector diffractometer
Absorption correction: multi-scan (*SADABS*; Sheldrick, 1996 ▶) *T*_min_ = 0.915, *T*_max_ = 0.960
6372 measured reflections
2606 independent reflections
1798 reflections with *I* > 2σ(*I*)
*R*_int_ = 0.098

#### Refinement

*R*[*F* ^2^ > 2σ(*F* ^2^)] = 0.069
*wR*(*F* ^2^) = 0.216
*S* = 1.08
2606 reflections
198 parameters
3 restraints
H atoms treated by a mixture of independent and constrained refinement
Δρ_max_ = 0.39 e Å^−3^
Δρ_min_ = −0.40 e Å^−3^

### 

Data collection: *APEX2* (Bruker, 2004 ▶); cell refinement: *SAINT* (Bruker, 2004 ▶); data reduction: *SAINT*; program(s) used to solve structure: *SHELXS97* (Sheldrick, 2008 ▶); program(s) used to refine structure: *SHELXL97* (Sheldrick, 2008 ▶); molecular graphics: *SHELXTL* (Sheldrick, 2008 ▶); software used to prepare material for publication: *SHELXL97*.

## Supplementary Material

Crystal structure: contains datablock(s) I, global. DOI: 10.1107/S1600536812045175/ld2073sup1.cif

Structure factors: contains datablock(s) I. DOI: 10.1107/S1600536812045175/ld2073Isup2.hkl

Supplementary material file. DOI: 10.1107/S1600536812045175/ld2073Isup3.cml

Additional supplementary materials:  crystallographic information; 3D view; checkCIF report

## Figures and Tables

**Table 1:** Hydrogen-bond geometry (Å, °)

*D*—H⋯*A*	*D*—H	H⋯*A*	*D*⋯*A*	*D*—H⋯*A*
O1—H1⋯N1	0.83 (6)	2.07 (9)	2.611 (5)	122 (7)
O1*W*—H1*W*⋯O1^i^	0.84 (4)	1.99 (4)	2.768 (5)	153 (5)
N1—H1*A*⋯O1*W*	0.90 (4)	2.20 (4)	3.012 (5)	150 (4)

Symmetry code: (i) 
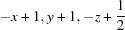
.

**Fig. 1.**
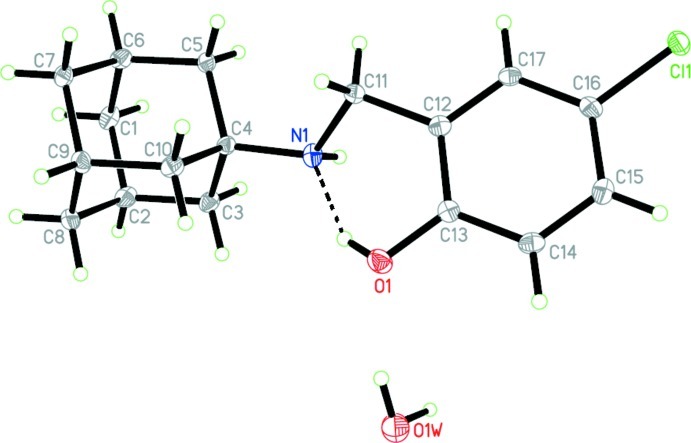
Molecular structure of the title compound with displacement ellipsoids drawn at 30% probability level.

**Fig. 2.**
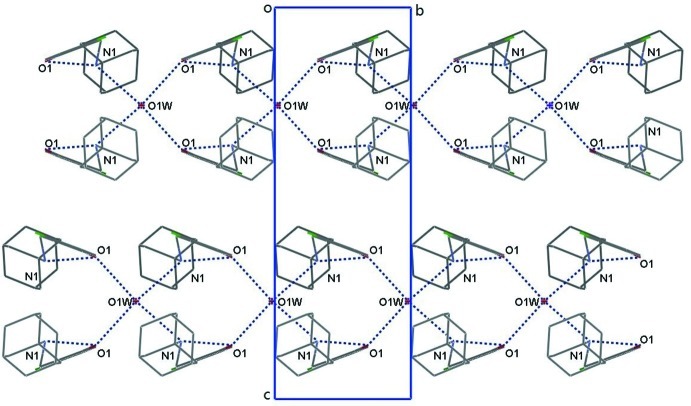
Packing diagram showing the H-bonded chains parallel to b axis. H atoms are omitted for clarity.

## supplementary crystallographic information

### Comment

Amantadine and its derivatives attract interest because of their biological activity and many potential applications (Camps *et al.*, 2008). As an extension of our previous work on the compounds containing an adamantane group, we synthesized the title compound by reduction of the corresponding Schiff base. It crystallizes with solvent water. Asymmetric unit contains one molecule of the title compound and one half of solvent water (Fig. 1). In the organic molecule, all bond lengths and angles are normal and comparable with another reported compound, *N*-(2-Hydroxybenzyl)adamantan-1-amine (Wang *et al.*, 2012). The hydroxyl O atom is involved in hydrogen bond (Table 1) with amino N atom with the OH···N distance of 2.611 (5) Å. This intra-molecular hydrogen bond formally results in a chiral center at the nitrogen atom, but the centrosymmetric crystal represents a racemate. The intramolecular hydrogen bond forms a *R*^2^_1_(6) ring which stabilizes the molecular conformation (Table 1). In the crystal, the couples of organic molecules are alternated with crystallization water molecules along *b* axis forming intermolecular O(W)—H···O and N—H···O(W) hydrogen bonds (Fig. 2).

### Experimental

Amantadine hydrochloride (0.376 g, 2.0 mmol) and KOH (0.112 g, 2.0 mmol) were stirred in 10 ml of anhydrous alcohol for 2 h. The produced white precipitate was filtered out and the transparent filtrate was added dropwise to 5-chloro-2-hydroxybenzaldehyde (0.312 g, 2.0 mmol) in 10 ml of anhydrous alcohol under constant stirring. The resulting solution was refluxed for *ca*. 3 h, concentrated to about 5 ml through reduced pressure distillation and then left at room temperature. A yellow Schiff base precipitate was obtained after one week under slow solvent evaporation.

NaBH_4_ (0.303 g, 8 mmol) was added into solution of the Schiff base (0.580 g, 2 mmol) in anhydrous methanol (10 ml). After 1 h stirring, a light-yellow solid, 4-chloro-2-((adamantan-1-ylamino)methyl)phenol was filtered and dried. A crystal of the title compound suitable for X-ray analysis has developed from a solution in H_2_O/EtOH mixture (1:2 *v*/*v*) after 5 days of slow solvent evaporation.

### Refinement

The C-bound H atoms were positioned geometrically with C—H = 0.93–0.98 Å, and treated as riding with *U*_iso_(H) = 1.2 *U*_eq_(C). H atoms bonded to N and O atoms were located in difference Fourier series and refined isotropically.

### Crystal data

**Table:**

C_17_H_22_ClNO·0.5H_2_O	*F*(000) = 1288
*M**_r_* = 300.82	*D*_x_ = 1.347 Mg m^−^^3^
Monoclinic, *C*2/*c*	Mo *K*α radiation, λ = 0.71073 Å
Hall symbol: -C 2yc	Cell parameters from 941 reflections
*a* = 25.469 (16) Å	θ = 3.3–22.6°
*b* = 6.365 (4) Å	µ = 0.26 mm^−^^1^
*c* = 18.306 (11) Å	*T* = 296 K
β = 91.815 (12)°	Block, yellow
*V* = 2966 (3) Å^3^	0.35 × 0.30 × 0.16 mm
*Z* = 8	

### Data collection

**Table:**

Bruker APEXII CCD area-detector diffractometer	2606 independent reflections
Radiation source: fine-focus sealed tube	1798 reflections with *I* > 2σ(*I*)
Graphite monochromator	*R*_int_ = 0.098
φ and ω scans	θ_max_ = 25.0°, θ_min_ = 2.2°
Absorption correction: multi-scan (*SADABS*; Sheldrick, 1996)	*h* = −30→28
*T*_min_ = 0.915, *T*_max_ = 0.960	*k* = −7→7
6372 measured reflections	*l* = −13→21

### Refinement

**Table:**

Refinement on *F*^2^	Primary atom site location: structure-invariant direct methods
Least-squares matrix: full	Secondary atom site location: difference Fourier map
*R*[*F*^2^ > 2σ(*F*^2^)] = 0.069	Hydrogen site location: inferred from neighbouring sites
*wR*(*F*^2^) = 0.216	H atoms treated by a mixture of independent and constrained refinement
*S* = 1.08	*w* = 1/[σ^2^(*F*_o_^2^) + (0.0716*P*)^2^ + 4.2412*P*] where *P* = (*F*_o_^2^ + 2*F*_c_^2^)/3
2606 reflections	(Δ/σ)_max_ = 0.001
198 parameters	Δρ_max_ = 0.39 e Å^−^^3^
3 restraints	Δρ_min_ = −0.40 e Å^−^^3^

### Special details

**Table:**

Geometry. All esds (except the esd in the dihedral angle between two l.s. planes) are estimated using the full covariance matrix. The cell esds are taken into account individually in the estimation of esds in distances, angles and torsion angles; correlations between esds in cell parameters are only used when they are defined by crystal symmetry. An approximate (isotropic) treatment of cell esds is used for estimating esds involving l.s. planes.
Refinement. Refinement of F^2^ against ALL reflections. The weighted R-factor wR and goodness of fit S are based on F^2^, conventional R-factors R are based on F, with F set to zero for negative F^2^. The threshold expression of F^2^ > 2sigma(F^2^) is used only for calculating R-factors(gt) etc. and is not relevant to the choice of reflections for refinement. R-factors based on F^2^ are statistically about twice as large as those based on F, and R- factors based on ALL data will be even larger.

### Fractional atomic coordinates and isotropic or equivalent isotropic displacement parameters (Å^2^)

**Table:**

	*x*	*y*	*z*	*U*_iso_*/*U*_eq_	
C1	0.68802 (17)	0.9819 (7)	0.1855 (2)	0.0245 (10)	
H1C	0.6718	1.0857	0.2164	0.029*	
H1D	0.7254	1.0115	0.1847	0.029*	
C2	0.67926 (18)	0.7615 (7)	0.2166 (2)	0.0276 (11)	
H2	0.6948	0.7526	0.2662	0.033*	
C3	0.61919 (16)	0.7207 (7)	0.2187 (2)	0.0225 (10)	
H3A	0.6128	0.5828	0.2392	0.027*	
H3B	0.6030	0.8246	0.2495	0.027*	
C4	0.59505 (16)	0.7331 (6)	0.1410 (2)	0.0181 (9)	
C5	0.60470 (15)	0.9535 (6)	0.1103 (2)	0.0194 (9)	
H5A	0.5884	1.0577	0.1410	0.023*	
H5B	0.5890	0.9646	0.0615	0.023*	
C6	0.66397 (17)	0.9950 (7)	0.1079 (2)	0.0222 (10)	
H6	0.6700	1.1354	0.0879	0.027*	
C7	0.68971 (16)	0.8294 (6)	0.0594 (2)	0.0194 (9)	
H7A	0.6748	0.8377	0.0101	0.023*	
H7B	0.7271	0.8560	0.0575	0.023*	
C8	0.70416 (16)	0.5981 (7)	0.1683 (3)	0.0256 (10)	
H8A	0.6985	0.4590	0.1882	0.031*	
H8B	0.7417	0.6221	0.1671	0.031*	
C9	0.68029 (16)	0.6101 (6)	0.0909 (2)	0.0217 (10)	
H9	0.6967	0.5042	0.0601	0.026*	
C10	0.62049 (16)	0.5697 (7)	0.0927 (2)	0.0215 (10)	
H10A	0.6052	0.5776	0.0435	0.026*	
H10B	0.6140	0.4301	0.1117	0.026*	
C11	0.50199 (15)	0.7241 (6)	0.0828 (2)	0.0195 (9)	
H11A	0.4970	0.8739	0.0758	0.023*	
H11B	0.5173	0.6669	0.0393	0.023*	
C12	0.45058 (16)	0.6210 (6)	0.0955 (2)	0.0182 (9)	
C13	0.44999 (15)	0.4119 (6)	0.1226 (2)	0.0190 (9)	
C14	0.40219 (17)	0.3142 (7)	0.1333 (2)	0.0225 (10)	
H14	0.4017	0.1768	0.1506	0.027*	
C15	0.35494 (16)	0.4183 (7)	0.1187 (2)	0.0220 (9)	
H15	0.3230	0.3522	0.1263	0.026*	
C16	0.35647 (16)	0.6241 (7)	0.0923 (2)	0.0196 (9)	
C17	0.40353 (16)	0.7250 (6)	0.0811 (2)	0.0184 (9)	
H17	0.4037	0.8626	0.0640	0.022*	
Cl1	0.29758 (4)	0.75941 (17)	0.07489 (6)	0.0262 (4)	
H1	0.5251 (19)	0.367 (15)	0.133 (6)	0.16 (5)*	
H1A	0.5178 (18)	0.748 (7)	0.181 (2)	0.045 (16)*	
N1	0.53801 (14)	0.6858 (6)	0.14802 (19)	0.0207 (8)	
O1	0.49570 (13)	0.3128 (5)	0.13804 (18)	0.0272 (8)	
O1W	0.5000	1.0204 (8)	0.2500	0.0532 (17)	
H1W	0.491 (2)	1.097 (7)	0.286 (2)	0.040 (16)*	

### Atomic displacement parameters (Å^2^)

**Table:**

	*U*^11^	*U*^22^	*U*^33^	*U*^12^	*U*^13^	*U*^23^
C1	0.025 (2)	0.030 (2)	0.019 (2)	−0.0099 (19)	0.0030 (17)	−0.0067 (19)
C2	0.027 (2)	0.038 (3)	0.017 (2)	−0.005 (2)	−0.0043 (18)	0.005 (2)
C3	0.021 (2)	0.028 (2)	0.019 (2)	−0.0037 (18)	0.0025 (17)	0.0021 (18)
C4	0.017 (2)	0.015 (2)	0.022 (2)	−0.0023 (16)	0.0028 (16)	0.0003 (17)
C5	0.019 (2)	0.020 (2)	0.020 (2)	0.0036 (17)	0.0064 (16)	−0.0012 (17)
C6	0.029 (2)	0.016 (2)	0.022 (2)	−0.0015 (18)	0.0032 (18)	−0.0026 (18)
C7	0.019 (2)	0.020 (2)	0.020 (2)	−0.0029 (17)	0.0056 (16)	−0.0023 (17)
C8	0.015 (2)	0.025 (2)	0.036 (3)	−0.0031 (18)	0.0000 (18)	0.006 (2)
C9	0.020 (2)	0.018 (2)	0.028 (3)	0.0019 (17)	0.0057 (17)	−0.0041 (18)
C10	0.025 (2)	0.018 (2)	0.021 (2)	−0.0047 (18)	0.0019 (17)	−0.0019 (17)
C11	0.018 (2)	0.022 (2)	0.019 (2)	0.0006 (17)	−0.0007 (16)	0.0023 (17)
C12	0.024 (2)	0.018 (2)	0.013 (2)	0.0003 (17)	−0.0001 (16)	−0.0017 (16)
C13	0.021 (2)	0.021 (2)	0.016 (2)	0.0025 (17)	0.0014 (16)	−0.0028 (17)
C14	0.030 (2)	0.015 (2)	0.022 (2)	−0.0027 (18)	−0.0010 (18)	0.0008 (18)
C15	0.021 (2)	0.029 (2)	0.016 (2)	−0.0024 (18)	−0.0002 (16)	−0.0037 (18)
C16	0.022 (2)	0.026 (2)	0.011 (2)	0.0057 (18)	−0.0022 (16)	−0.0013 (17)
C17	0.024 (2)	0.018 (2)	0.013 (2)	0.0010 (17)	0.0009 (16)	0.0015 (16)
Cl1	0.0204 (6)	0.0357 (7)	0.0225 (6)	0.0044 (5)	0.0000 (4)	0.0045 (5)
N1	0.0196 (18)	0.029 (2)	0.0139 (19)	0.0023 (16)	−0.0018 (14)	−0.0034 (15)
O1	0.0271 (18)	0.0216 (16)	0.0326 (19)	0.0043 (14)	−0.0043 (14)	0.0000 (14)
O1W	0.037 (3)	0.017 (3)	0.106 (6)	0.000	0.012 (3)	0.000

### Geometric parameters (Å, º)

**Table:**

C1—C6	1.531 (6)	C8—H8B	0.9700
C1—C2	1.533 (6)	C9—C10	1.546 (6)
C1—H1C	0.9700	C9—H9	0.9800
C1—H1D	0.9700	C10—H10A	0.9700
C2—C8	1.517 (6)	C10—H10B	0.9700
C2—C3	1.553 (6)	C11—C12	1.489 (6)
C2—H2	0.9800	C11—N1	1.502 (5)
C3—C4	1.534 (6)	C11—H11A	0.9700
C3—H3A	0.9700	C11—H11B	0.9700
C3—H3B	0.9700	C12—C17	1.387 (6)
C4—N1	1.493 (5)	C12—C13	1.421 (6)
C4—C10	1.523 (6)	C13—O1	1.346 (5)
C4—C5	1.534 (5)	C13—C14	1.387 (6)
C5—C6	1.534 (6)	C14—C15	1.392 (6)
C5—H5A	0.9700	C14—H14	0.9300
C5—H5B	0.9700	C15—C16	1.397 (6)
C6—C7	1.539 (6)	C15—H15	0.9300
C6—H6	0.9800	C16—C17	1.381 (6)
C7—C9	1.532 (6)	C16—Cl1	1.750 (4)
C7—H7A	0.9700	C17—H17	0.9300
C7—H7B	0.9700	N1—H1A	0.90 (2)
C8—C9	1.524 (6)	O1—H1	0.83 (2)
C8—H8A	0.9700	O1W—H1W	0.847 (19)
			
C6—C1—C2	109.6 (3)	C9—C8—H8A	109.6
C6—C1—H1C	109.8	C2—C8—H8B	109.6
C2—C1—H1C	109.8	C9—C8—H8B	109.6
C6—C1—H1D	109.8	H8A—C8—H8B	108.1
C2—C1—H1D	109.8	C8—C9—C7	109.4 (3)
H1C—C1—H1D	108.2	C8—C9—C10	109.7 (3)
C8—C2—C1	110.0 (4)	C7—C9—C10	109.0 (3)
C8—C2—C3	109.3 (4)	C8—C9—H9	109.6
C1—C2—C3	108.5 (4)	C7—C9—H9	109.6
C8—C2—H2	109.7	C10—C9—H9	109.6
C1—C2—H2	109.7	C4—C10—C9	109.6 (3)
C3—C2—H2	109.7	C4—C10—H10A	109.7
C4—C3—C2	109.5 (3)	C9—C10—H10A	109.7
C4—C3—H3A	109.8	C4—C10—H10B	109.7
C2—C3—H3A	109.8	C9—C10—H10B	109.7
C4—C3—H3B	109.8	H10A—C10—H10B	108.2
C2—C3—H3B	109.8	C12—C11—N1	108.9 (3)
H3A—C3—H3B	108.2	C12—C11—H11A	109.9
N1—C4—C10	110.2 (3)	N1—C11—H11A	109.9
N1—C4—C5	112.6 (3)	C12—C11—H11B	109.9
C10—C4—C5	109.7 (3)	N1—C11—H11B	109.9
N1—C4—C3	105.8 (3)	H11A—C11—H11B	108.3
C10—C4—C3	109.7 (3)	C17—C12—C13	119.7 (4)
C5—C4—C3	108.8 (3)	C17—C12—C11	121.3 (4)
C4—C5—C6	109.7 (3)	C13—C12—C11	119.1 (3)
C4—C5—H5A	109.7	O1—C13—C14	121.2 (4)
C6—C5—H5A	109.7	O1—C13—C12	119.6 (4)
C4—C5—H5B	109.7	C14—C13—C12	119.3 (4)
C6—C5—H5B	109.7	C13—C14—C15	121.1 (4)
H5A—C5—H5B	108.2	C13—C14—H14	119.4
C1—C6—C5	109.2 (3)	C15—C14—H14	119.4
C1—C6—C7	109.5 (3)	C14—C15—C16	118.6 (4)
C5—C6—C7	109.7 (3)	C14—C15—H15	120.7
C1—C6—H6	109.5	C16—C15—H15	120.7
C5—C6—H6	109.5	C17—C16—C15	121.4 (4)
C7—C6—H6	109.5	C17—C16—Cl1	119.2 (3)
C9—C7—C6	109.4 (3)	C15—C16—Cl1	119.4 (3)
C9—C7—H7A	109.8	C16—C17—C12	119.9 (4)
C6—C7—H7A	109.8	C16—C17—H17	120.0
C9—C7—H7B	109.8	C12—C17—H17	120.0
C6—C7—H7B	109.8	C4—N1—C11	118.0 (3)
H7A—C7—H7B	108.2	C4—N1—H1A	123 (4)
C2—C8—C9	110.2 (3)	C11—N1—H1A	96 (4)
C2—C8—H8A	109.6	C13—O1—H1	124 (7)
			
C6—C1—C2—C8	59.0 (5)	C5—C4—C10—C9	60.1 (4)
C6—C1—C2—C3	−60.5 (4)	C3—C4—C10—C9	−59.4 (4)
C8—C2—C3—C4	−59.4 (4)	C8—C9—C10—C4	59.3 (4)
C1—C2—C3—C4	60.6 (4)	C7—C9—C10—C4	−60.5 (4)
C2—C3—C4—N1	178.3 (3)	N1—C11—C12—C17	134.1 (4)
C2—C3—C4—C10	59.5 (4)	N1—C11—C12—C13	−46.0 (5)
C2—C3—C4—C5	−60.5 (4)	C17—C12—C13—O1	−178.6 (4)
N1—C4—C5—C6	177.4 (3)	C11—C12—C13—O1	1.5 (6)
C10—C4—C5—C6	−59.5 (4)	C17—C12—C13—C14	0.9 (6)
C3—C4—C5—C6	60.5 (4)	C11—C12—C13—C14	−179.0 (4)
C2—C1—C6—C5	60.9 (4)	O1—C13—C14—C15	178.8 (4)
C2—C1—C6—C7	−59.2 (4)	C12—C13—C14—C15	−0.7 (6)
C4—C5—C6—C1	−60.7 (4)	C13—C14—C15—C16	0.4 (6)
C4—C5—C6—C7	59.3 (4)	C14—C15—C16—C17	−0.4 (6)
C1—C6—C7—C9	59.9 (4)	C14—C15—C16—Cl1	−178.9 (3)
C5—C6—C7—C9	−59.9 (4)	C15—C16—C17—C12	0.7 (6)
C1—C2—C8—C9	−59.3 (4)	Cl1—C16—C17—C12	179.2 (3)
C3—C2—C8—C9	59.7 (4)	C13—C12—C17—C16	−0.9 (6)
C2—C8—C9—C7	59.7 (4)	C11—C12—C17—C16	179.0 (4)
C2—C8—C9—C10	−59.8 (4)	C10—C4—N1—C11	−72.3 (4)
C6—C7—C9—C8	−59.8 (4)	C5—C4—N1—C11	50.5 (5)
C6—C7—C9—C10	60.1 (4)	C3—C4—N1—C11	169.2 (3)
N1—C4—C10—C9	−175.5 (3)	C12—C11—N1—C4	166.7 (3)

### Hydrogen-bond geometry (Å, º)

**Table:**

*D*—H···*A*	*D*—H	H···*A*	*D*···*A*	*D*—H···*A*
O1—H1···N1	0.83 (6)	2.07 (9)	2.611 (5)	122 (7)
O1*W*—H1*W*···O1^i^	0.84 (4)	1.99 (4)	2.768 (5)	153 (5)
N1—H1*A*···O1*W*	0.90 (4)	2.20 (4)	3.012 (5)	150 (4)

Symmetry code: (i) −*x*+1, *y*+1, −*z*+1/2.
